# Phospholipase A2 enhances the cytotoxic activity of membranolytic peptide *Polybia* MP1 in some breast epithelial cells

**DOI:** 10.1016/j.bbrep.2026.102823

**Published:** 2026-10-02

**Authors:** Arindam Pramanik, Dapeng Wang, Dagmara Kobza, Lewis P. Reed, Paul A. Beales, Thomas A. Hughes

**Affiliations:** a Amity Institute of Biotechnology, Amity University, Noida, Uttar Pradesh, India; b Shandong Key Laboratory of Intelligent Oil & Gas Industrial Software, Qingdao Institute of Software, College of Computer Science and Technology, China University of Petroleum (East China), Qingdao, China; c Department of Pathology, Military Institute of Medicine, National Research Institute, Warsaw, Poland; d School of Health Sciences, York St. John University, York, UK; e School of Chemistry and Astbury Centre for Structural Molecular Biology, University of Leeds, Leeds, UK; f School of Medicine, University of Leeds, Leeds, UK

**Keywords:** Membranolytic peptide, Membrane-acting, Cancer therapy, Breast cancer

## Abstract

MP1, a peptide from the venom of the wasp *Polybia paulista*, has been investigated for anti-microbial and anti-cancer properties. The peptide binds and disrupts external-facing lipid membranes of various cell types, thereby reducing cellular viability, with some reported selectivity for cancer cells. The molecular basis of selectivity is poorly understood. We have assessed MP1 activity in a panel of human cells, with the aims of establishing the types most suited to development of future MP1-derived therapies and identifying molecular factors influencing responses.

Cellular viability was tested in 64 cancer cell lines after treatment with MP1 to determine IC_50_ values. These varied by 8.5-fold across the panel, with blood cancer lines proving most sensitive and colorectal cancer lines most resistant, and breast cells showing the greatest variance. Transcriptome data were downloaded for cell lines and relationships between expression levels of each individual gene and IC_50_ values were assessed using linear regression. 67 genes demonstrated significant correlations after correction for multiple testing, within which lipases were significantly over-represented. The impact on viability of exogenous phospholipase A2 with and without MP1 was tested in four different breast epithelial cell lines that represented a wide range of intrinsic sensitivities to MP1 alone. Phospholipase A2 had little significant influence on viability alone, but caused potent and dose-dependent potentiation of MP1-induced cytotoxicity in two of the four cell lines tested.

We conclude that phospholipase A2 and MP1 act together on some breast cancer cell membranes, and that this combinatorial influence could impact on future development of MP1 as a cancer therapy.

## Highlights

• MP1 reduced viability in 64 cancer cell lines, with IC_50_ values varying 8.5-fold across the panel.
• Expression of lipases in these cell lines was significantly associated with MP1 response.
• Exogenous phospholipase A2 enhances MP1-induced cytotoxicity in some breast cell types.

## 1 Introduction

Membranolytic peptides that occur naturally in venoms and toxic secretions from various organisms have been studied extensively as potential anti-microbial [1] or anti-cancer agents [2]. This potential is based on their ability to bind and disrupt external-facing biological membranes, typically with some selectivity for certain membrane characteristics, thereby reducing the viability of their target cells. In the main, the complex molecular interactions that underpin membrane selectivity are not understood and require further study for the medicinal potential of the agents to be fully realized. Membrane features that have been confirmed as influencing sensitivity to specific peptides include lipid composition [3,4], membrane curvature [5], membrane charge [6], and the presence of specific molecular cofactors [7], although these features are seldom independent of each other and therefore the key determinants remain unclear.

We have recently demonstrated that MP1, a peptide purified from the venom of the wasp *Polybia paulista*, has potent but varied membranolytic activity against a range of human cell types [8]. Sensitivity to MP1 varies by at least 6-fold across different cell types [9], with some reports of a degree of specificity for cancer cells [10,11] leading to the idea that MP1 could be developed as a cancer therapeutic [11,12]. Membrane sensitivity to MP1 has been shown to depend on the inter-related features of membrane viscosity and stiffness [13], lipid composition [10,[14], [15], [16]] and membrane charge [17]. The majority of this work has been in the context of purified model membranes, although some findings are based on bacteria or, rarely, mammalian cells. Here, we focused initially on determining the activity of MP1 across different human cancer cell lines, with the aims of both determining which cancer types might be most suitable for treatment with future MP1-derived therapies [9,12], and identifying determinants of relative sensitivity or resistance in the context of human cells. With respect to determinants of response, transcriptional profiles are potentially upstream of the direct biological determinants and therefore we correlated gene expression with MP1 sensitivity in a panel of cell lines, an approach that has been used previously in high throughput screens to identify response determinants for many established cancer drugs [18,19]. We demonstrated that expression of endogenous lipases correlated with MP1 sensitivity. This merited further investigation since phospholipases have evident potential for direct action on the phospholipid membrane, and because phospholipases have themselves been identified as components of venoms that act in synergy with some membranolytic peptides [[20], [21], [22], [23]], although this has not previously been demonstrated for *Polybia* MP1.

Here, we present the first comprehensive assessment of MP1 sensitivities across a large panel of human cell lines, and an investigation of candidate determinants of sensitivity, leading to identification of potent potentiation of MP1 activity by phospholipase A2.

## 2 Methods and materials

### 2.1 Cell culture and viability assays

Determination of MP1 sensitivities in 64 cancer cell lines (see Table 1) was performed by Crown Bioscience (Beijing, China). In brief, cells were cultured at 37^°^C in humidified 5% CO_2_/air using standard media appropriate for the cell types supplemented with 10-20% fetal bovine serum (Thermo Fisher Scientific or Sigma-Aldrich; both Shanghai, China). Cells were seeded in media at 4x10^3^ cells/well in technical triplicates in flat- and clear-bottomed black-walled 96-well plates (Corning, New York, USA). Cells were treated for 48 h with MP1 (IDWKKLLDAAKQIL; synthesised by Bio-Synthesis, Lewisville, TX, USA) at highest doses of 316 μM, and with 3.16-fold serial dilutions to achieve 8 further lower doses, the lowest being 31.8 nM. Viable cells were quantified using CellTiter-Glo Luminescent Assays (Promega, Madison, WI, USA) and an EnVision Multi Label plate-reader (PerkinElmer, Shelton, CT, USA) according to the manufacturers' protocols. This assay was selected as it requires a minimum number of experimental manipulations before reading the result and was therefore particularly well suited to high throughput analyses. Dose-response data were analysed relative to untreated control, and IC_50_ values were determined by fitting non-linear regression models with sigmoidal dose responses in Prism v5 (GraphPad, Boston, MA, USA). AU565, MDA-MB-468, BT-474 and MCF-10A cells were purchased from American Type Culture Collection (ATCC) for further assays and were validated for lack of mycoplasma contamination (MycoAlert; Lonza, Basel, Switzerland) and identity (short tandem repeat profiling; Source Bioscience, Nottingham, UK). Cells, except MCF-10A, were cultured in Dulbecco's Modified Eagle Medium (DMEM) supplemented with 10% fetal calf serum and 100 units/ml penicillin-streptomycin (Thermo Scientific, Waltham, MA, USA) at 37°C in humidified 5% CO_2_/air. MCF-10A was cultured in DMEM supplemented with 5% horse serum, 0.1 μg/mL cholera toxin, 0.5 μg/ml hydrocortisone, 0.02 μg/mL epidermal growth factor, and 100 units/ml penicillin−streptomycin (Thermo Scientific, Waltham, MA, USA) at 37°C in humidified 5% CO_2_/air. Cells were maintained and experiments were performed under conditions ensuring cell densities that supported exponential growth. MTT assays were used to assess survival/proliferation as previously reported [24], and as is common practice within the field for viability assays performed manually in low to medium throughput. In brief, cells were seeded in technical triplicates in 96-well plates at 1x10^4^ cells/well in complete growth media and incubated for 18 h. Cells were treated with up to 42 μM MP1, up to 0.5 μM phospholipase A2 (PLA2; purified from porcine pancreas; Sigma-Aldrich catalogue number P6534 [≥600 units/mg protein], Gillingham, UK), or the combination for 24 h. Cells were then incubated with 0.5 mg/ml MTT (Sigma-Aldrich, Gillingham, UK) dissolved in PBS for 3 h. Formazan crystals were dissolved using 200 μl isopropanol and absorbance was measured at 570 nm on a microplate spectrophotometer (Biotek Instruments, Winooski, VT, USA).

**Table 1:** Cancer cell lines have varied MP1 sensitivities. 64 cancer cell lines were treated for 48 h with a range of doses of MP1 and viable cells were quantified to allow calculation of IC_50_ doses. Cell lines are listed, numbered 1-64 in two columns, with the IC_50_ values.

#	Cell line	Tissue of origin	IC_50_ (μM)	#	Cell line	Tissue of origin	IC_50_ (μM)
1	CCRF-CEM	Blood	27.30	33	ACHN	Kidney	30.71
2	HL-60	Blood	10.57	34	UO.31	Kidney	25.46
3	K-562	Blood	19.52	35	A549	Lung	38.62
4	Molt-4	Blood	28.12	36	A-427	Lung	26.50
5	RPMI 8226	Blood	23.10	37	Calu-3	Lung	33.48
6	A-172	Brain/neuronal	50.24	38	DMS 114	Lung	45.47
7	LN-229	Brain/neuronal	22.24	39	NCI-H226	Lung	22.73
8	M059K	Brain/neuronal	37.28	40	NCI-H23	Lung	23.72
9	SF268	Brain/neuronal	25.26	41	NCI-H460	Lung	18.39
10	SH-SY5Y	Brain/neuronal	42.42	42	NCI-H522	Lung	29.69
11	U251	Brain/neuronal	29.90	43	EBC-1	Lung	34.84
12	U-87 MG	Brain/neuronal	32.28	44	Caov-3	Ovary	47.86
13	AU565	Breast	20.51	45	OVCAR-3	Ovary	49.76
14	BT-20	Breast	25.13	46	OVCAR-4	Ovary	24.53
15	BT-474	Breast	90.04	47	OVCAR-5	Ovary	30.73
16	BT-549	Breast	27.48	48	OVCAR-8	Ovary	24.72
17	CAL-120	Breast	42.23	49	SK-OV-3	Ovary	44.13
18	Hs 578T	Breast	34.26	50	SW626	Ovary	37.47
19	MCF7	Breast	30.53	51	A2780cis	Ovary	27.38
20	T47D	Breast	30.64	52	AsPC-1	Pancreas	32.73
21	SK-BR-3	Breast	32.98	53	BxPC-3	Pancreas	24.93
22	Caco-2	Colorectal	35.16	54	Capan-1	Pancreas	63.74
23	COLO 205	Colorectal	78.44	55	Capan-2	Pancreas	41.30
24	DLD-1	Colorectal	34.36	56	PANC-1	Pancreas	55.44
25	HCT 116	Colorectal	30.62	57	DU 145	Prostate	18.85
26	HCT-15	Colorectal	32.14	58	PC-3	Prostate	34.82
27	HT-29	Colorectal	67.02	59	Malme-3M	Skin	30.72
28	LoVo	Colorectal	65.02	60	SK-MEL-28	Skin	19.03
29	LS 174T	Colorectal	40.10	61	SK-MEL-5	Skin	14.95
30	LS411 N	Colorectal	34.12	62	A2058	Skin	26.92
31	786-O	Kidney	20.30	63	A-375	Skin	30.02
32	A498	Kidney	24.33	64	SR	Thymus	40.82

### 2.2 On-line data mining and computational analyses

Transcriptome data were downloaded from the Cancer Cell Line Encyclopedia (https://sites.broadinstitute.org/ccle) for the following 54 cell lines in December 2019: HL-60, K-562, RPMI 8226, LN-229, M059K, SF268, U-87 MG, AU565, BT-20, BT-549, CAL-120, Hs 578T, MCF7, SK-BR-3, HCT 116, HCT-15, HT-29, LoVo, 786-O, ACHN, A549, Calu-3, DMS 114, NCI-H226, NCI-H23, NCI-H460, NCI-H522, Caov-3, OVCAR-4, OVCAR-8, SK-OV-3, AsPC-1, BxPC-3, Capan-1, Capan-2, PANC-1, DU 145, PC-3, Malme-3M, SK-MEL-28, SK-MEL-5, LS411 N, EBC-1, A2058, A-375, A-172, U251, T47D, A498, UO.31, OVCAR-3, SR, Caco-2, A-427. Data were not available for the remaining 10 lines from this resource on the date accessed. Expression data included expression levels of the 22939 genes that were detectable in at least one of the cell lines. Linear regression analyses were performed in R to assess associations between gene expression (Y) and IC50 values for MP1 (X) using the model Y = a+bX. Regression coefficients and corresponding p values were obtained for each gene. P values were adjusted for multiple testing using the Benjamini–Hochberg method. Over-representation analyses were performed using the WEB-based GEne SeT AnaLysis Toolkit (http://www.webgestalt.org) in Feb 2020 using the 67 genes identified in Table 2 as the input and the entire 22939 genes expressed in at least one cell line as the reference set.

**Table 2:** Expression of 67 genes correlates significantly with MP1 sensitivity. genes. Relationships across 54 cell lines between expression levels of 22939 individual genes and the IC_50_ values for MP1 were assessed using linear regression, and significance was determined after correction for multiple testing. Genes showing significance of p < 0.05 (Benjamini–Hochberg adjusted) are listed in two columns, along with the p values.

Gene name	BH p value	Gene name	BH p value
HOXB5	0.00589398	HOXB-AS4	0.0274898
RP11-120K24.3	0.00952302	PDE4C	0.0274898
GIPC2	0.0159483	AC008982.2	0.0274898
IHH	0.0159483	CHDH	0.0290928
HOXA-AS3	0.0159483	HOTTIP	0.03316773
HOXA6	0.0159483	TTC22	0.0334018
HOXB6	0.0159483	RP11-112J3.16	0.0334018
HOXB-AS3	0.01762261	RP11-255B23.4	0.03420214
HOXA11-AS	0.01935167	RP13-122B23.8	0.03420214
BMP4	0.01935167	LIPG	0.03420214
RP11-927P21.2	0.01935167	FAAH	0.03715168
CTD-2619J13.3	0.01935167	RP1-170O19.20	0.03715168
TRIM15	0.02111917	C11orf53	0.03715168
NYNRIN	0.02271355	MIR192	0.03775745
RP11-465B22.8	0.0232999	GPX2	0.03775745
GRHL3	0.0232999	RP11-299G20.2	0.03775745
PIGHP1	0.0232999	RP11-304L19.3	0.03775745
TRIM31-AS1	0.02373949	CTB-39G8.3	0.03775745
TRIM10	0.02373949	HOXB-AS2	0.03775745
KLF5	0.02373949	SLCO1B7	0.04166385
MFSD4	0.0274898	ZCCHC14	0.04166385
CAPN8	0.0274898	RSPH1	0.04166385
AC013268.5	0.0274898	S100A5	0.0417744
AC112229.7	0.0274898	AC004067.5	0.0417744
MIR3131	0.0274898	HOTAIRM1	0.0417744
SOWAHB	0.0274898	RP11-395P17.3	0.0417744
CCAT1	0.0274898	MUC5AC	0.0417744
ZNF214	0.0274898	CAPN12	0.0417744
PLA2G16	0.0274898	FLNB-AS1	0.04275389
LIPT2	0.0274898	UGT1A13P	0.04652797
COLCA2	0.0274898	NPFFR1	0.04652797
AL163953.2	0.0274898	RP11-304L19.1	0.04652797
AKAP13	0.0274898	C15orf62	0.04971366
LYSMD4	0.0274898		

### 2.3 Statistical analyses

Unless stated otherwise above, statistical analyses were performed using Prism v9 or v10 for Windows (GraphPad, Boston, MA, USA) and the tests mentioned in the figure legends.

## 3 Results

### 3.1 Cancer cell lines have varied MP1 sensitivities that do not differ consistently by tissue of origin

We have previously shown that cell lines have varied sensitivities to the membranolytic peptide MP1 [9]. In order to understand the basis of this variation, we greatly expanded our assessment of relative sensitivities to encompass 64 human cancer cell lines derived from diverse tissues of origin (see Table 1). Cells were treated with a range of MP1 doses for 48 h and survival was assessed using luminescent viability assays. Responses are summarised as IC_50_ values (Table 1), while primary data are shown for two representative cell lines in Fig. S1. IC_50_ values varied by 8.5-fold across the panel, with HL-60 cells, a promyelocytic leukemia line, being the most sensitive and BT-474 cells, a breast cancer line, being most resistant. Breast epithelial lines showed the greatest variance in IC_50_ values within a group, demonstrating that sensitivity was modifiable even within cell types of the same origin. Cell lines derived from blood cells and from colorectal cells showed the lowest and highest mean IC_50_ values respectively, and the difference between these groups was statistically significant (p < 0.04; Fig. 1). However, this was the only significant difference noted between the tissues of origin, and overall the tissue groups were characterised by substantial variation between individual lines and little consistent pattern.

**Fig. 1:**
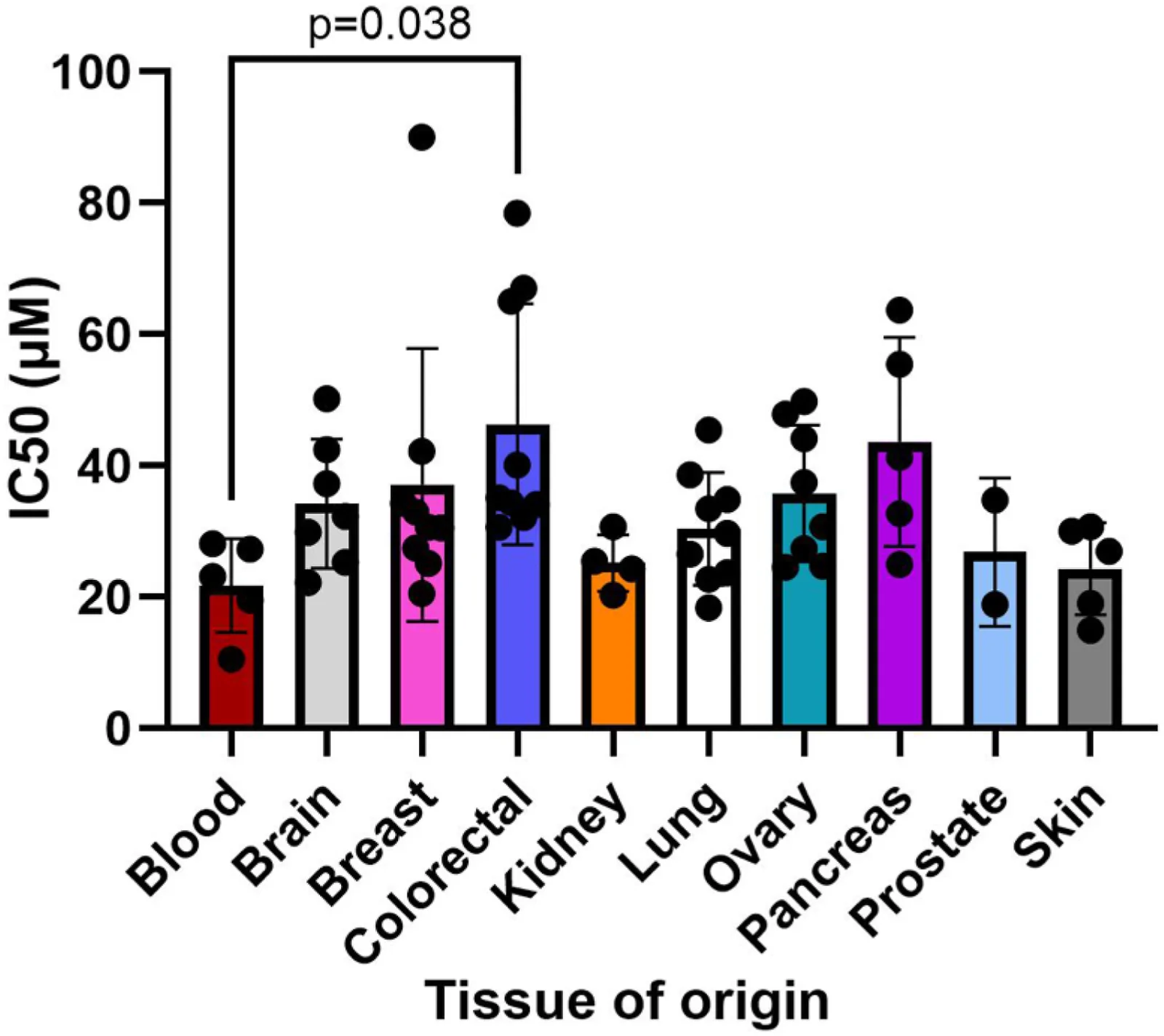
Cancer cell lines have varied MP1 sensitivities that do not differ consistently by tissue of origin. 64 cancer cell lines derived from 11 different tissues of origin were treated for 48 h with a range of doses of MP1 and viable cells were quantified to allow calculation of IC_50_ doses. Cell lines were grouped according to tissue of origin and IC_50_ values for individual lines are shown (black circles) within the group (mean and standard deviation). The one cell line from thymus (SR cells) was excluded. Differences between groups were assessed using the Kruskal-Wallis test, and the only significant difference is shown.

### 3.2 Expression of 67 genes correlates significantly with MP1 sensitivity

In order to understand determinants of MP1 sensitivity in these cell lines, we aimed to identify correlations between relative expression levels of genes and IC_50_ values. Transcriptome data derived from RNA-sequencing were available for 54 of the cell lines from the Cancer Cell Line Encyclopedia. Expression data were curated to include only genes with detectable expression in at least one cell line, leaving a total of 22939 genes. Relationships between expression levels of each individual gene and the IC_50_ values were assessed using linear regression, and statistical significance was corrected for multiple testing using the Benjamini-Hochberg method. A total of 67 genes demonstrated significant correlations (Table 2; or see Table S1 for the full analyses).

Commonality in function of these genes was assessed using over-representation analyses, identifying enrichment of genes in 10 gene ontology groups using a relatively permissive, exploratory, statistical threshold of FDR<0.15 (Table 3). This permissive threshold was used because groups were not identified at more stringent levels. These groups fell into three categories: developmental processes (four groups, sharing four core genes: BMP4, HOXB5, HOXB6, IHH), T cell biology (five groups, all of which matched only the same two genes: BMP4, IHH), and lipase activity (one group with three genes matching only this group: FAAH, LIPG, PLA2G16). MP1 is known to act directly on the external surface of the plasma membrane, and therefore over-represented gene categories were assessed to identify those with plausible direct mechanisms of action at the membrane. The developmental and T cell biology groups almost exclusively contained genes encoding signaling ligands and transcription factors that do not obviously have direct influences on plasma membrane function. By contrast, the lipase activity group was of interest since genes within this category could potentially directly influence membrane composition and stability through actions on constituent lipids. For example, proteins with phospholipase A2 (PLA2) activity, which make up more than one fifth of the human genes in this ontology group, have been widely reported to have direct membranolytic activity [20,25] and to act in synergy with a range of membranolytic peptides in the context of venoms [23]. PLA1 proteins also have similar, although lesser roles for some venoms [26,27]. Therefore, we concluded the over-representation of the lipase gene ontology group supported the hypothesis that lipases overall may have a role in determining MP1 sensitivity.

**Table 3:** 10 gene ontology groups are over-represented within the genes that showed correlations with MP1 sensitivity. The 67 genes listed in Table 2 were assessed for over-representation of gene ontology (GO) groups. Groups showing over-representation (FDR<0.15) are named, along with their GO reference and a list of matching genes. Genes in italics matched within multiple over-represented groups.

Gene ontology (GO) name	GO reference	FDR	Matching genes (italics indicates appears in multiple groups)
Skeletal system development	GO:0001501	0.121	AKAP13, *BMP4, HOXA6, HOXB5, HOXB6, IHH*
Pattern specification process	GO:0007389	0.115	*GRHL3, BMP4, HOXA6, HOXB5, HOXB6, IHH*
Embryonic organ morphogenesis	GO:0048562	0.115	*GRHL3, BMP4, HOXB5, HOXB6, IHH*
Embryonic skeletal system development	GO:0048706	0.015	*BMP4, HOXA6, HOXB5, HOXB6, IHH*
Negative regulation of T cell differentiation in thymus	GO:0033085	0.115	*BMP4, IHH*
Regulation of immature T cell proliferation	GO:0033083	0.115	*BMP4, IHH*
Regulation of immature T cell proliferation in thymus	GO:0033084	0.116	*BMP4, IHH*
Immature T cell proliferation	GO:0033079	0.116	*BMP4, IHH*
Immature T cell proliferation in thymus	GO:0033080	0.116	*BMP4, IHH*
Lipase activity	GO:0016298	0.140	FAAH, LIPG, PLA2G16

### 3.3 Exogenous PLA2 increases MP1 sensitivity in some breast cancer cell lines

Having noted that lipases, including a PLA2 family member (PLA2G16), had been identified as potential determinants of MP1 sensitivity, we were interested to test whether overall exogenous PLA2 activity could impact directly on cellular sensitivity to MP1, as previously reported for some membranolytic peptides [23]. Note that our hypothesis was not that the three specific genes identified within the lipase activity gene ontology group individually define MP1 sensitivity, rather that activities within the wider ontology group identified were potentially of interest, with the previous literature on exogenous PLA2 guiding us to test this specifically. Assessment and manipulation of the wider gene ontology group builds on the strength of over-representation analysis in highlighting other genes that may not have been individually significant although may still be functionally important, and is in line with previous studies that identified key regulators and direct therapeutic targets by looking beyond individually significant genes, and investigating other members of the gene ontology groups identified in over-representation analyses [[28], [29], [30]]. We have previously studied MP1 sensitivity extensively in breast epithelial cell lines [9], and in addition our screening here showed that breast epithelial cells spanned a particularly wide-range of sensitivities (Fig. 1); therefore, we used breast lines with the aim of testing cell lines representing both relative resistance and sensitivity. We selected four representative cell lines: AU565, which is relatively sensitive to MP1, BT-474 and MCF-10A, which are highly resistant (see Table 1 and [9]), and MDA-MB-468, which has an intermediate sensitivity [9]. Cells were treated with a range of doses of purified PLA2, with different doses of MP1, or with the combinations, and survival was assessed using MTT assays after 24 h (Fig. 2).

**Fig. 2:**
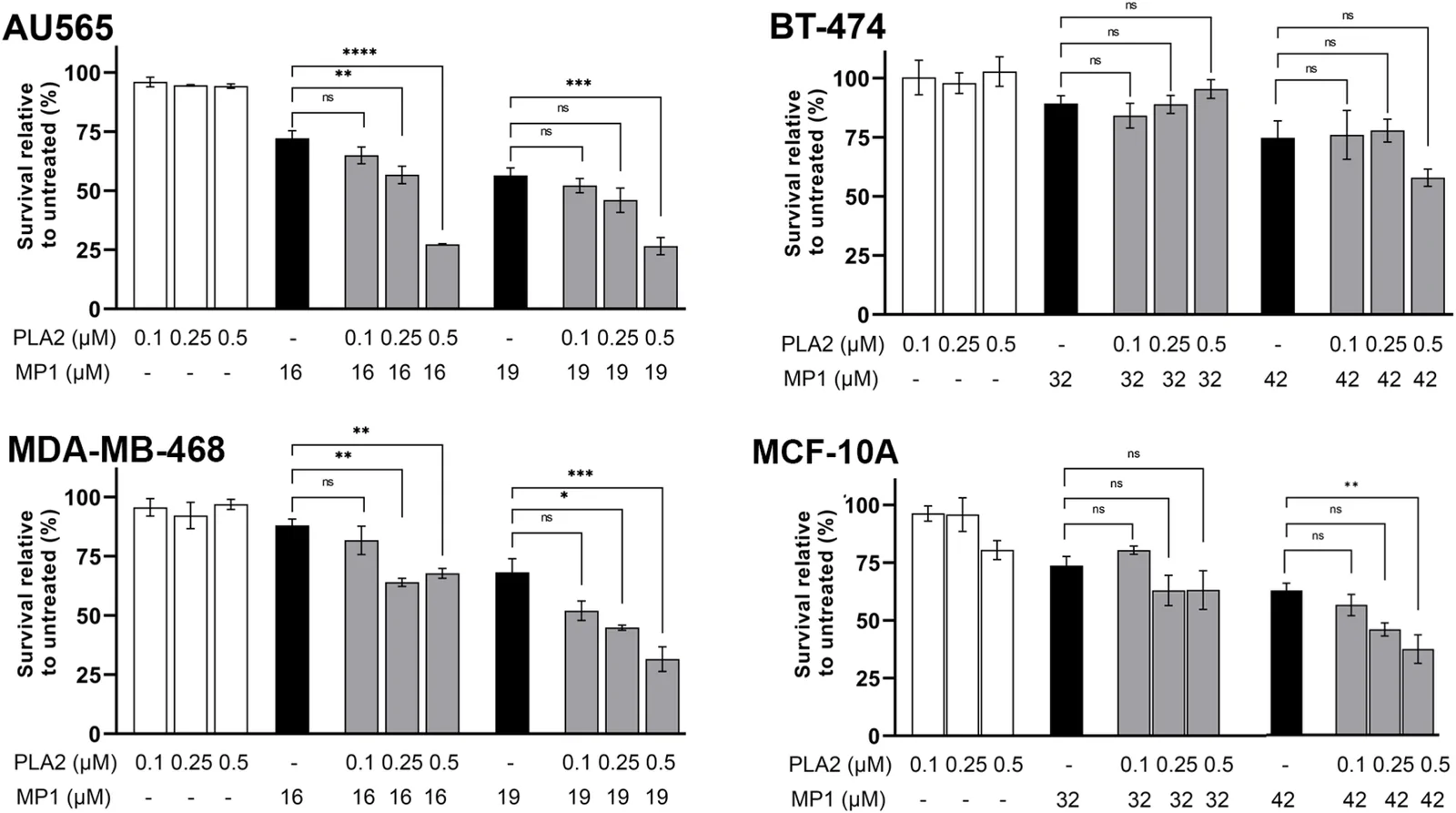
Exogenous PLA2 increases MP1 sensitivity in some breast cancer cell lines. AU565 (top left), MDA-MB-468 (bottom left), BT-474 (top right), or MCF-10A cells (bottom right) were treated for 24 h with control, purified PLA2 (open bars), MP1 (black bars), or the combination (grey bars) at the doses stated. Viable cells were quantified using MTT assays. Data are shown relative to untreated and represent means ( ± standard error) of two biological replicates (each of which was a mean derived from technical triplicates). Statistical significance was assessed using 2-way ANOVA with Tukey's multiple comparisons tests to adjust p values to account for multiple testing.

PLA2 alone had little influence on survival (open bars), except in one cell line (MCF-10A) and at only the highest dose, and did not show a dose-response. As expected, MP1 alone caused dose- and cell line-dependent reductions in cell survival (black bars). For example, AU565 cells showed survival reduced to 72.3% and 56.6% at the two MP1 doses used (16 and 19 μM respectively), while the more resistant MDA-MB-468 cells showed 88.1% and 68.2% survival at these doses. Since BT-474 and MCF10A cells were known to be much more resistant, we used higher MP1 doses for these lines (32 and 42 μM), reducing survival to 74.8% or 64.5% respectively at the higher dose. In both AU565 and MDA-MB-468 cells, every combination treatment caused greater reductions in survival than MP1 alone (grey bars; p < 0.05 in 7 out of 9 cases). The reduction in survival associated with the combination also exceeded the additive effect of both single agents in every case. This was especially marked with the highest doses of PLA2 (0.5 μM); for example, survival of AU565 after treatment with the combinations was only 27.4% or 26.6%, while individual doses of PLA2 reduced survival to 94.3% and of MP1 to 72.3% (16 μM) or 56.6% (19 μM). It was also notable that PLA2 consistently showed a dose-response when added in combination with MP1, which was absent using PLA2 alone. By contrast in BT-474 cells, PLA2 combined with MP1 did not cause significant reductions in survival compared to MP1 alone at any dose tested (grey bars). The result in MCF-10A cells was more mixed, with the effects of the combination treatment just exceeding the additive effect of both single agents when MP1 was used at the higher concentration, and this was significant at the highest dose of PLA2. We concluded that PLA2 acts to potentiate the influence of MP1 on membranes in breast cancer cells that are sensitive to MP1, but potentially less so in those that are relatively resistant.

## 4 Discussion

The literature concerning relative sensitivities of different human cells to *Polybia* MP1 consists of only a few studies, each of which includes small numbers of cell types. Our analysis in Table 1 is by far the largest published. Our observation that blood cancer cell lines are relatively sensitive to MP1 (Fig. 1) is in line with the absolute sensitivities shown in the two published papers using leukemia cells [10,31], although these do not show comparisons between the leukemic and any other cancer lines. The literature also supports our conclusion that cancer cells show widely varied sensitivities, however many authors have focused more on comparisons between cancer cells and fibroblasts, demonstrating the cancer cells are more sensitive. This includes comparisons between prostate or lung cancer cells and human primary fibroblasts [11,32] and leukemia, bladder, prostate and breast cancer cells and various immortalized fibroblast lines [8,31,33]. In the context of our findings regarding PLA2, it is interesting to note that fibroblasts reportedly have relatively low basal levels of secreted PLA2 enzymes, which are induced in fibroblasts in a range of pathologies [[34], [35], [36]]. We speculate that these low basal levels contribute to the reported relative resistance of fibroblasts *in vitro* to MP1.

The strategy we used to identify determinants of MP1 sensitivity, namely by correlating transcriptome data with IC_50_ values, is an increasingly common method. In our case, we produced and used the only available systematic dataset of IC_50_ values for our agent of interest, whereas for many therapeutics these response data are available at web-portals including the Cancer Therapeutics Response Portal [37] or Genomics of Drug Sensitivity in Cancer [38], and extend to data from more than 800 cell lines. While the resources and overall approach are well-established, the specific methodology for assessing correlations and validating findings are hugely varied between studies. We have used simple regression analyses for assessing correlations, as have others [39,40], however a variety of methodologies can be used including integration of additional data sets [41,42], such as somatic mutations and proteome. Regardless of discovery methodology, validation remains key. We have undertaken confirmatory functional experiments in cell lines (Fig. 2), while in the context of clinically-approved agents others have validated by demonstrating their gene or gene signature correlates with actual response to treatment in samples from patients [43,44].

Synergy in terms of membrane-disruption and cell death between PLA2 and other membranolytic peptides has been reported previously for toxins from three cobra species (*N. nigricollis*, *N. melanoleuca* and *N. mossambica)* and for melittin from the honeybee (*Apis mellifera*) [23], but not for *Polybia* MP1. Importantly, we do not claim and have not tested in our validation that the three specific genes identified within the lipase activity gene ontology group define MP1 sensitivity, and the lack of this assessment is a limitation of our study. As an alternative, we find that exogenous PLA2 can influence this sensitivity. Mechanisms underlying the synergy with these other toxins remain unclear, with reports suggesting melittin and PLA2 bind each other to form a complex with enhanced PLA2 activity [45], or that melittin induces membrane structural deformities on which PLA2 acts with increased processivity [20]. This latter model provides a framework to interpret our observations that BT-474 cells both are relatively resistant to MP1 *and* do not demonstrate potentiation with MP1 and PLA2, since a threshold level of MP1 activity may be required to induce the deformities on which PLA2 would act. Co-operativeity between PLA2 and MP1 is of particular interest in the cancer field, given efforts to harness MP1 as a potential cancer therapeutic [9,11,12]. Secreted PLA2s are upregulated and play a protumorigenic role in various cancer types [46] including breast [47] although, conversely, tumour suppressor roles have been shown in cancers of the gastrointestinal tract. It would be expected that protumorigenic upregulation of secreted PLA2s would sensitise cancer cells specifically to MP1, and therefore may widen the therapeutic window for its potential use in these particular cancer types.

## CRediT authorship contribution statement

**Arindam Pramanik:** Writing – review & editing, Formal analysis, Data curation, Conceptualization. **Dapeng Wang:** Writing – review & editing, Formal analysis, Data curation. **Dagmara Kobza:** Writing – review & editing, Formal analysis, Data curation. **Lewis P. Reed:** Writing – review & editing, Formal analysis, Data curation. **Paul A. Beales:** Writing – review & editing, Supervision, Project administration, Funding acquisition, Formal analysis, Data curation, Conceptualization. **Thomas A. Hughes:** Writing – review & editing, Writing – original draft, Supervision, Project administration, Funding acquisition, Formal analysis, Data curation, Conceptualization.

## Ethics approval and consent to participate

Not applicable.

## Funding

This research was supported by the UK Engineering and Physical Sciences Research Council (EPSRC): EP/R03608X/1. DW was supported by the Taishan Scholars Program of Shandong Province (tsqn202312110) and the Fundamental Research Funds for the Central Universities (25CX06001A).

## Declaration of competing interest

The authors declare no conflict of interest.

## Data Availability

Data are available within the manuscript/supplementary information or can be requested directly from the communicating authors.
