# Supplementary material for: Phospholipase A2 enhances the cytotoxic activity of membranolytic peptide Polybia MP1 in some breast epithelial cells
Source: Biochem Biophys Rep. 2026 Oct 2;48:102823. doi: 10.1016/j.bbrep.2026.102823 (PMC13661229; doi:10.1016/j.bbrep.2026.102823)
Supplement: Supplementary file 1 [file mmc2.docx]

**Figure S1.** Cell lines show varied dose-responses to MP1. HL-60 or SW626 cells were treated for 48 h with a range of doses of MP1 and viable cells were quantified. Data are shown relative to untreated control and represent means (+/- standard deviations) of technical triplicates.

*Excel spreadsheet attachment*

**Table S1.** Relationships across 54 cell lines between expression levels of 22939 individual genes and the IC_50_ values for MP1 were assessed using linear regression, and significance was determined as a simple p value (column E), and after correction for multiple testing using the Benjamini–Hochberg method (column F).
